# The trend of dental check-up and prevalence of dental complications following the use of bone modifying agents in patients with metastatic breast and prostate cancer: analysis of data from the Korean National Health Insurance Service

**DOI:** 10.1186/s12913-024-10859-7

**Published:** 2024-04-02

**Authors:** Ah Reum Lim, Wonse Park, Seok Joo Moon, Min Sun Kim, Soohyeon Lee

**Affiliations:** 1 Division of Medical Oncology, Department of Internal Medicine, Korea University Anam Hospital, Korea University College of Medicine, 73, Goryeodae-ro, Seongbuk-gu, 02841 Seoul, Korea; 2 Department of Advanced General Dentistry, College of Dentistry, Yonsei University, Seoul, Korea; 3 Department of Biostatistics, Korea University College of Medicine, Seoul, Korea

**Keywords:** Metastatic breast cancer, Metastatic prostate cancer, Bone metastasis, Dental check-up, Medication-related osteonecrosis of the jaw, Bisphosphonate osteonecrosis of the jaw, Bone modifying agents, Bone targeted agents

## Abstract

**Background:**

Bone-modifying agents (BMA) are key components in the management of cancer patients with bone metastasis. Despite their clinical benefits, the use of BMA is associated with dental adverse events (AEs) including medication-related osteonecrosis of the jaw (MRONJ). This study investigated the frequency of dental surveillance before BMA treatment and the prevalence of dental AEs including MRONJ, after BMA treatment in patients with bone metastasis from breast and prostate cancer using data from the national health insurance system.

**Methods:**

Data, including age, cancer diagnosis, administered BMA, and dental AEs during cancer treatment, of patients with bone metastasis from breast and prostate cancer who received at least one infusion of BMA between 2007 and 2019 were extracted from the Korean National Health Insurance Service (KNHIS) dataset.

**Results:**

Of the 15,357 patients who received BMA, 1,706 patients (11.1%) underwent dental check-ups before BMA treatment. The proportion of patients receiving dental check-up increased from 4.4% in 2007 to 16.7% in 2019. Referral to dentists for a dental check-up was more active in clinics/primary hospitals than general/tertiary hospitals, and medical doctors and urologists actively consulted to dentists than general surgeons, regardless of the patient’s health insurance status. After BMA treatment, 508 patients (3.8%) developed dental AEs, including abscess (42.9%), acute periodontitis (29.7%), acute pericoronitis (14.9%), and MRONJ (12.5% of dental AEs cases, 0.5% of total BMA treated patients).

**Conclusions:**

Considering the long treatment period in patients with metastatic cancer, coordination between dentists and oncologists is necessary to ensure appropriate dental management before the initiation of BMA.

**Supplementary Information:**

The online version contains supplementary material available at 10.1186/s12913-024-10859-7.

## Background

The use of bone-modifying agents (BMA), such as bisphosphonates and receptor activation of nuclear factor kappa-B (RANK) ligand inhibitors, in patients with bone metastases from breast, prostate cancer, and multiple myeloma, is associated with improvements in morbidity, pain, quality of life, and skeletal-related events (SREs) [1–6]. BMA is also recommended in patients with advanced lung cancer, renal cancer, and other solid tumors with bone metastases, particularly those at high risk of SREs and a life expectancy > 3 months [1, 2, 7].

Despite several clinical benefits, the use of BMA is associated with medication-related osteonecrosis of the jaw (MRONJ), which presents in approximately 1–9% of patients with advanced cancer [8–11]. MRONJ can occur spontaneously or following invasive dental procedures such as tooth extraction, and its signs and symptoms include exposure of the maxilla, pain due to inflammation of the exposed area, bleeding, osteonecrosis of the inferior alveolar canal and maxillary sinuses, and orodermal fistula. These complications seriously affect the patient’s quality of life and cancer treatment schedule [12].

With the advent of new treatments such as targeted therapy and immunotherapy, the overall survival and treatment period of cancer patients with bone metastases are prolonged. Accordingly, the cumulative BMA dose and period are lengthened, resulting in increasing dental AEs as well as MRONJ. Chronic dental infections such as periodontitis, periapical lesions, or pericoronitis caused by wisdom teeth can be converted into acute dental infections as the immune function weakens during cancer treatment. Acute dental infection can cause pain, swelling, and bleeding and sometimes lead to invasive treatment such as tooth extraction. This not only discontinues or delays chemotherapy but also worsens the patient’s quality of life [13].

The European Society for Medical Oncology (ESMO), the American Society for Clinical Oncology (ASCO), and Cancer Care Ontario (CCO) published guidelines on the use of BMA in metastatic bone disease and recommended that oral care assessment, including a comprehensive dental, periodontal, and oral radiographic examination should be undertaken before initiating therapy [1, 14, 15]. Many studies have established that preventive oral care methods combined with effective oral health practices are associated with a lower prevalence of MRONJ [16]. Therefore, patients should receive education regarding dental hygiene and MRONJ and undergo a comprehensive dental examination before starting BMA treatment [17]. During BMA use, regular follow-up with a dental professional should be encouraged, and invasive dental procedures should be avoided as possible [15].

However, there are few studies on the status of dental screening to prevent dental AEs before BMA treatment and the prevalence of dental complications including MRONJ after BMA treatment in real-world practice. We aimed to investigate the frequency of dental check-ups the presence of dental AEs including MRONJ during BMA treatment in patients with bone metastasis from breast and prostate cancer using claims data from the Korean National Health Insurance Service (KNHIS) database.

## Materials and methods

### Data source

The KNHIS was over 97% of the total Korean population. We used KNHIS claims data from January 2007 to December 2019. These data provide detailed information on demographics and healthcare utilization, including diagnostic codes (International Classification of Disease 10th revision, ICD-10), procedure codes, and prescriptions.

### Study population

We identified all newly diagnosed patients with breast and prostate cancer between January 2007 and December 2019 (ICD-10 codes: C50 and C61). We used a 6-month washout period to exclude cancer patients who had been diagnosed with bone metastasis in the past. Patients were followed up for at least 2 years and assessed for the development of bone metastases and dental AEs. Patients diagnosed with bone metastases were defined as those with at least three claims per year for the prescription of BMA, such as denosumab, zoledronic acid, and pamidronate, using drug prescription codes (ATC codes M05BX04, M05BA08, and M05BA03). Dental AEs were identified using the procedure codes for irrigation and drainage (U4454-U4457, U4464, U4467) and tooth extraction (U4411–U4417, U4420, UD620), and diagnostic codes for dental complications such as inflammatory conditions of the jaw (MRONJ, K102, and M871), acute periodontitis (K052, K0528, K0529), periodontal abscess of gingival origin (K0520, K0521), and acute pericoronitis (K0522) using ICD-10 codes. Dental check-ups were identified using the EDI codes for clinical oral evaluations (AA100, AA106, AA107, AA109 AA200, AA206, AA207, AA209), panoramic radiographic images (G9701), and procedure codes for scaling (U2233).

### Statistical analysis

Data on the prevalence of dental check-ups and dental AEs in each type of cancer and the basic demographic characteristics of the patients were obtained. Demographic characteristics were summarized as mean (standard deviation) and range (minimum, maximum) for continuous variables and frequency (percentage) for categorical variables. The prevalence of dental AEs was analyzed, and the annual prevalence was also presented. The annual prevalence of MRONJ (ICD-10 code K102) was investigated using the prescription codes for pamidronate and zoledronic acid. Proportions of dental care were summarized according to patients’ residential area, hospital type, and types of medical benefits. Chi-squared tests were performed to compare the proportions of complications and medical departments between the group that received dental care and the group that did not. All statistical analyses were performed using SAS software (version 9.4; SAS Institute Inc., Cary, NC, USA), and statistical significance was set at two-tailed *p* < 0.05.

## Results

### The trend of dental check-up before BMA administration

**Fig. 1:**
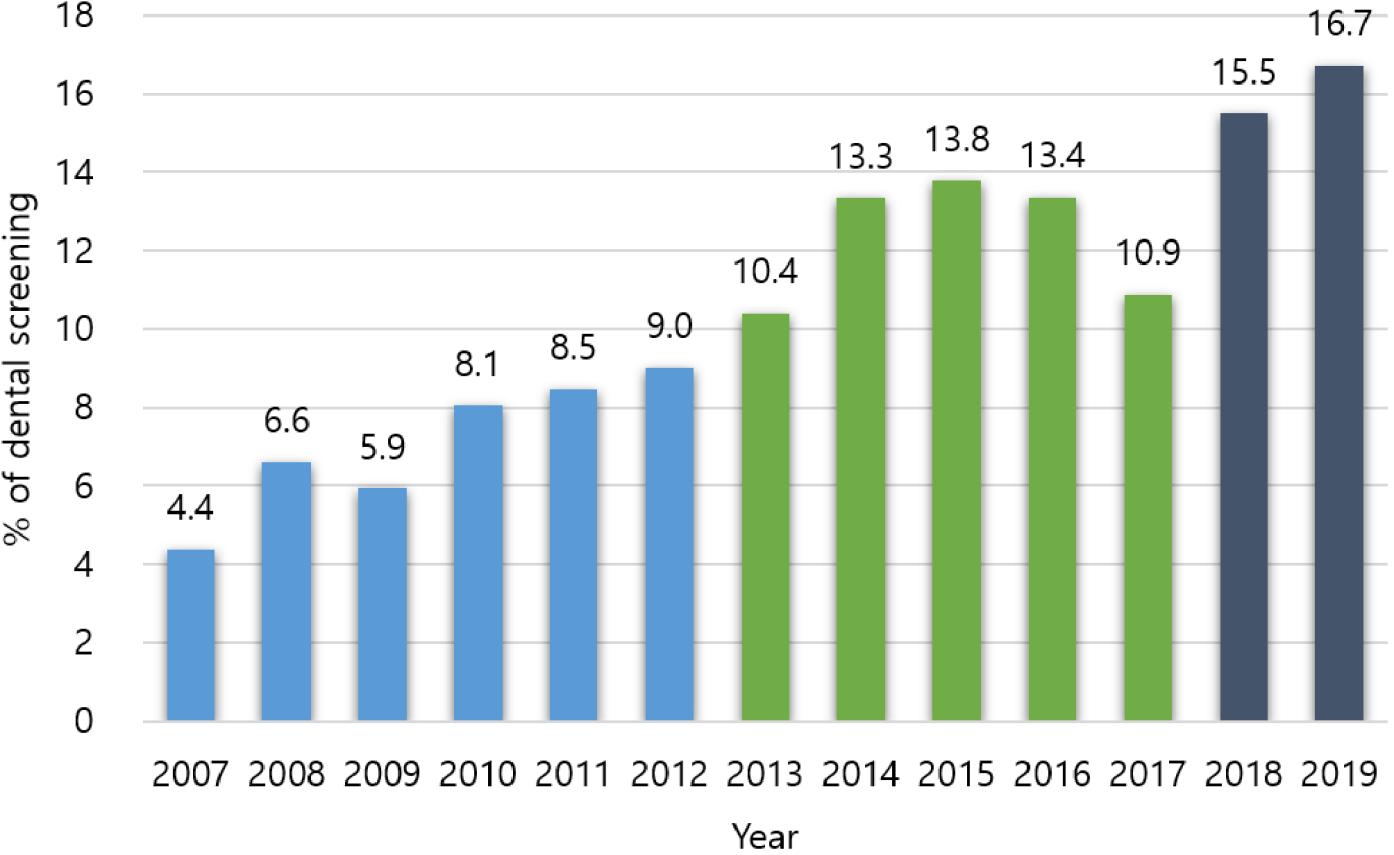
The trend of dental check-up before BMA treatment since 2007

A total of 15,357 patients with breast or prostate cancer who developed bone metastasis were treated with BMA between 2007 and 2019 (Additional Table 1). Of 15,357 patients, male and female patients were 3643 (23.7%) and 11,714 (76.2%), respectively. Among the male patients, 3,584 (98.4%) had prostate cancer, and 59 (1.6%) had breast cancer. The median ages of men and women were 72 years and 53 years, respectively (Table 1). According to the KNHIS database, the proportion of patients who received dental check-up among those treated with BMA for breast or prostate cancer with bone metastases increased from 3.1% in 2007 to 8.3% in 2015, temporarily decreased to 3.8% in 2016, and then increased again to 11.4% in 2018. Overall, of the 15,357 patients, 1,706 (11.1%) underwent dental check-up before BMA treatment. The proportion of patients undergoing dental check-up steadily increased from 4.38% in 2007 to 16.72% in 2019 (Fig. 1).

### Dental check-up frequency according to hospital type and treating physicians

We analyzed dental check-up status before BMA treatment based on the type of hospital, treating physician, and health insurance (Table 2). The proportion of dental surveillance before BMA treatment showed a difference according to the size of the hospital; it was the highest at clinics/primary hospitals (17.3%), followed by general hospitals (12.2%), and tertiary public hospitals (9.9%).

Further, according to the treating physician, there was a difference in the dental referral frequency —11.1% from medical doctors, 13.3% from urologists, and 9.3% from general surgeons. In terms of health insurance status, there was no significant difference in the proportion of patients who underwent dental check-ups between those covered by health insurance (11.1%) and others (medical care, homeless people, foreign workers [11.5%]).

**Fig. 2:**
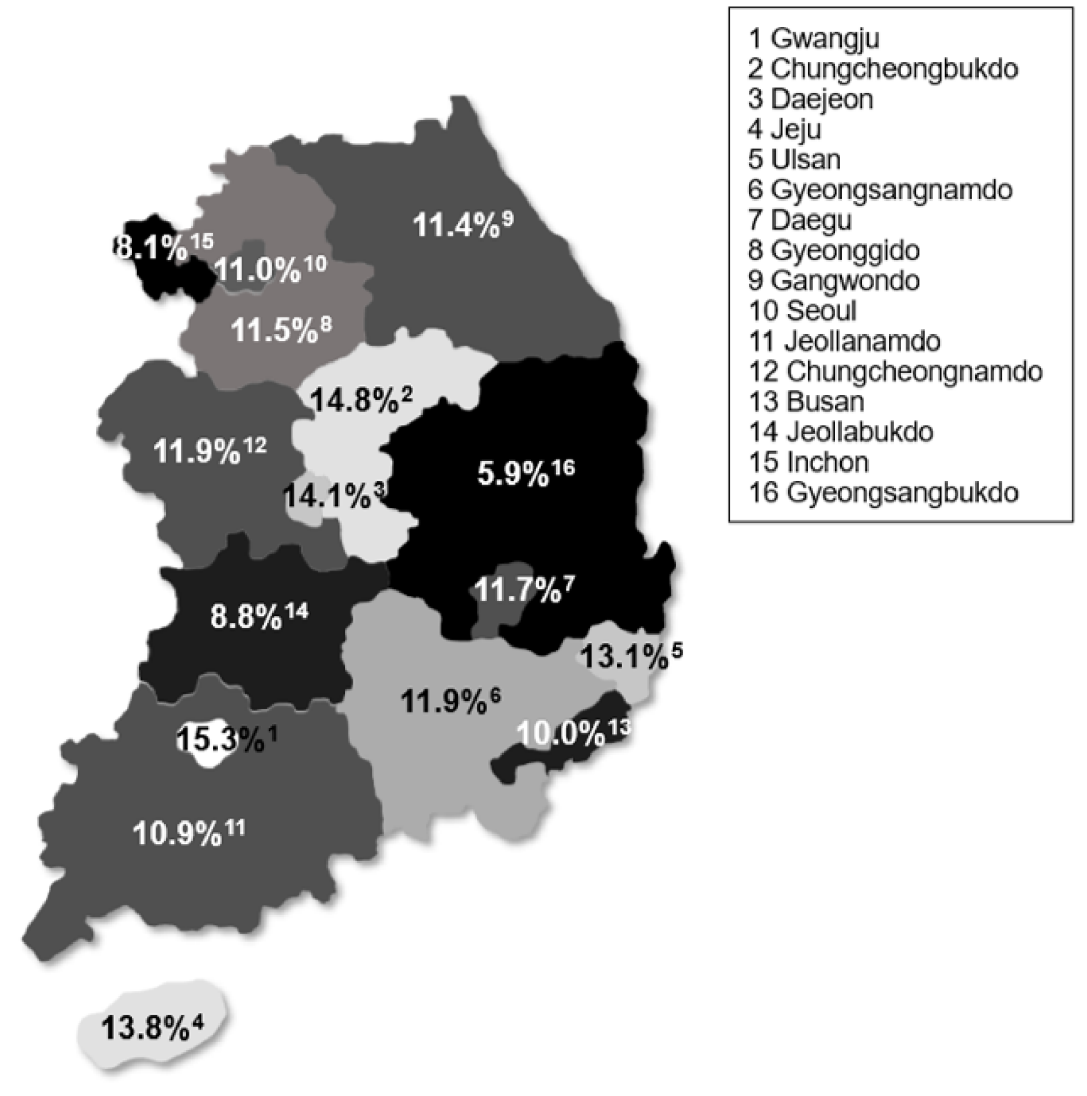
Dental check-up status before BMA treatment by region

The comparison of dental check-ups status by region showed that mid-sized cities, such as Gwangju (15.3%), Daejeon (14.1%), and Ulsan (13.1%), had a higher proportion of patients with dental check-ups before BMA treatment than Seoul (11.0%) and Inchon (8.1%). Jeollabuk-do (8.8%) and Gyeongsangbuk-do (5.9%) reported dental check-up frequency far below the average; thus, it is necessary to improve the distribution of medical resources and related services (Fig. 2).

**Table 1:** Baseline demographics of the study population

	Male	Female	Total
Age (median, min-max)	72 (31–100)	53 (17–94)	57 (17–100)
Cancer, *N* (%)			
Breast cancer	59 (1.62)	11,714 (100.00)	11,773 (76.66)
Prostate cancer	3,584 (98.38)	0 (0.00)	3,584 (23.34)

### Dental adverse events after BMA treatment

Dental AEs events were presented in 590 (3.8%) of 15,357 patients receiving BMA treatment. MRONJ was reported in 74 cases, accounting for 12.5% of all dental AEs and 0.5% of all patients receiving BMA. The MRONJ incidence was 91.1% with zoledronic acid and 8.9% with pamidronate. The mean time to MRONJ occurrence was 2396.4 days with zoledronic acid and 1306.9 days with pamidronate (Additional Table 2; Fig. 1). The MRONJ incidence in the study refers to MRONJ that occurred naturally, and that MRONJ incidence after extraction and dental procedures was not examined. Of 590 patients with dental AEs, periapical abscesses of gingival origin without sinus and acute periodontitis were presented in 223 and 175 patients, respectively. Periapical abscesses of gingival origin with sinus were presented in 30 cases, and acute pericoronitis was presented in 88 cases (Table 3).

**Fig. 3:**
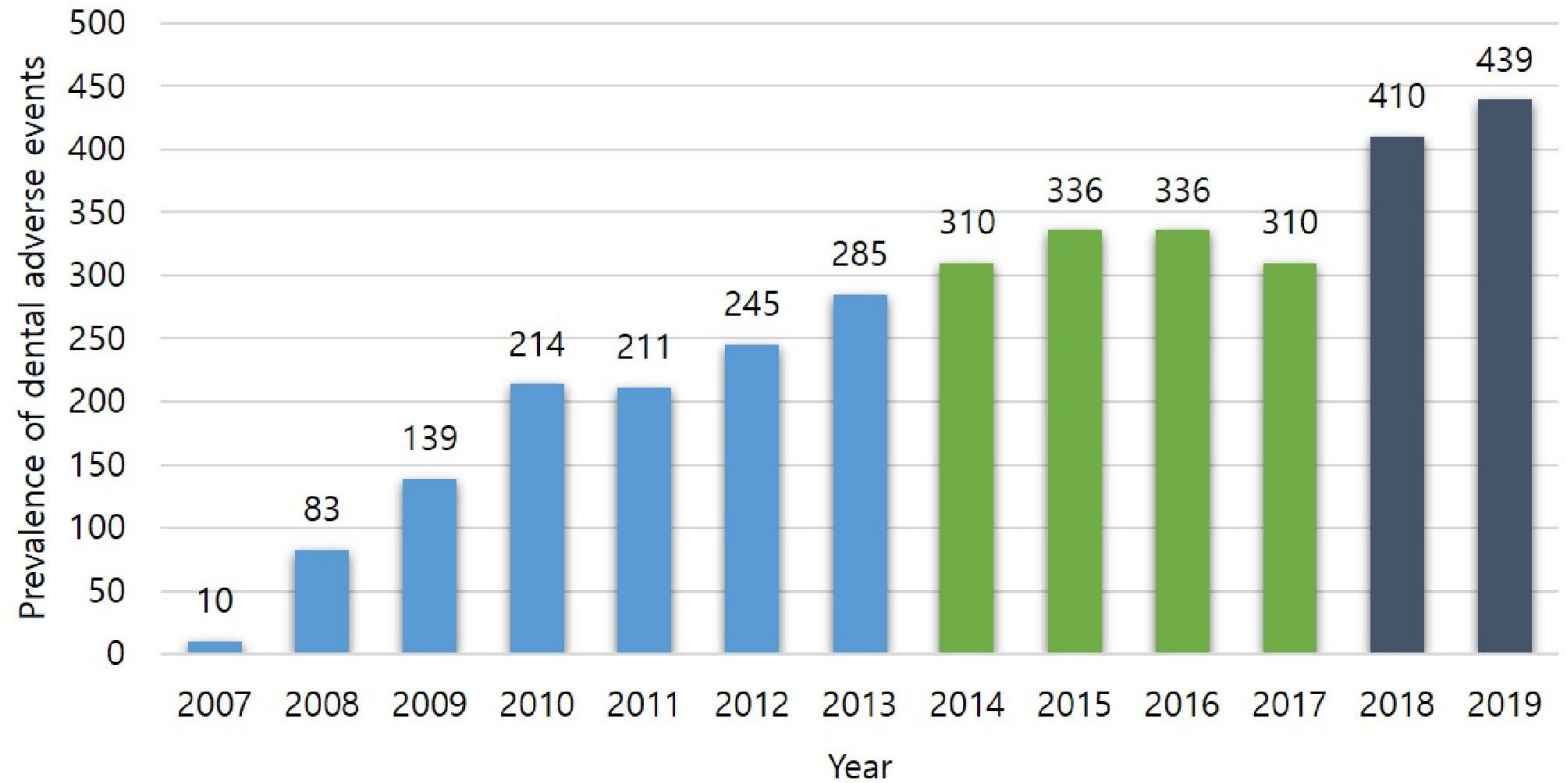
Prevalence of dental adverse events after BMA treatment since 2007

Figure 3 shows the prevalence of dental AEs after BMA treatment. In 2007, the initial stage of this analysis, the dental AEs might not have been sufficiently captured, but they have been increased every year (Fig. 3). Among 1,706 patients who underwent dental check-up before BMA treatment, 509 (29.8%) required dental intervention, whereas only 2919 (20.7%) of 13,651 patients who did not undergo dental surveillance required dental intervention (Table 4).

**Table 2:** Dental check-up by hospital type, department and health insurance

	Patient who receiving dental care, N	All patients, N	Proportion of dental check-up among all patients (%)
Hospital type			
Tertiary general hospital	976	9,858	9.9
General hospital	532	4,355	12.2
Hospital/clinic Department	198	1,144	17.3
Internal medicine	943	8,507	11.1
General surgery	405	4,379	9.3
Urology	170	1,277	13.3
Others	188	1,194	15.8
Health insurance type			
Health insurance	1,573	14,199	11.1
Others*	133	1,158	11.5

*Person for medical care, homeless people, foreign workers

**Table 3:** Incidence of adverse dental events during BMA treatment

Adverse dental events	N (%)
Periapical abscess from gingival origin	
Without sinus	223 (37.8)
With sinus	30 (5.1)
Acute periodontitis	175 (29.7)
Acute pericoronitis	88 (14.9)
Medication-related osteonecrosis of jaw	74 (12.5)
Total events	590 (100.0)

*BMA*, Bone-modifying agent

**Table 4:** Dental intervention after the initiation of BMAs

	Dental check-up (*N* = 1,706)	Without dental check-up (*N* = 13,651)	p-value*
Dental intervention, *N* (%)			< 0.0001
Yes	509 (29.8)	2,819 (20.7)	
No	1,197 (70.2)	10,832 (79.3)	

*BMA*, Bone-modifying agent

* p-value by chi-square test

## Discussion

New treatments, such as targeted therapies and immunotherapies, have led to a real transition in cancer survivorship. The emergence of a growing population of patients with metastatic cancer has raised issues about the unique needs and improvement of their care during cancer treatment. Dental health is one of the huge unmet needs during the long-term survival of metastatic cancer patients with bone metastasis. These patients should have their oral health checked by a dentist before beginning a BMA treatment. Considering that MRONJ, which is the most serious complication of BMA, is difficult to treat and stop or delay cancer treatment, dental prevention is even more important.

According to the KNHIS data, a nationwide Korea health service database system, from 2007 to 2019, MRONJ was reported in 74 cases, accounting for 0.5% of patients receiving BMA, which seemed to be lower [9, 10] or similar with 0– 0.019% in clinical trials [9, 18, 19]. A systemic review analyzed the prevalence of MRONJ after zoledronic acid administration was increased as 0.4–1.6%, 0.8–2.1%, and 1.0–2.3% after 1, 2, and 3 years of exposure, respectively [20]. Dental AEs were presented in 3,328 (21.7%) of the total 15,357 patients receiving BMA treatment, and most dental AEs included invasive dental procedures such as tooth extraction, incision, and drainage known as risk factors for MRONJ. From a preventive point of view, the frequency of dental check-ups before BMA treatment was 11.1%, but it showed a trend of increasing every year. The dental screening patterns differed depending on the treating physicians, region, and hospital size, but medical benefits were not affected. There are several potential reasons for the differences in the frequency of dental checks prior to BMA treatment by hospital size. While tertiary general hospitals may possess specialized departments, challenges such as scheduling and the narrow perspective of experts who focus only on their professional field might impede seamless collaboration between oncologists and dentists. In contrast to this, physicians at primary and secondary grade hospitals appear to be more active in recommending dental care, presumably because of the easier accessibility of patients to more dentistry. This suggests that tertiary general hospitals should prioritize enhancing collaboration efforts. Additionally, it was observed that the surgery department referred patients for dental checks less frequently compared to internal medicine and urology departments, indicating potential issues related to perception and education among different specialties. These findings underscore the importance of raising awareness and emphasizing the necessity of dental checks before initiating BMA treatment, especially among doctors in tertiary general hospitals and various departments. Although several guidelines recommend dental screening before BMA treatment [1, 15, 21, 22], dental screening was not actively performed during the whole study period. This might be related to the perception that anti-cancer therapy is more critical than dental surveillance and reluctant to the high cost of dental procedures or procedure-induced pain.

Although it is the first study to investigate the status of dental screening for cancer patients with bone metastases at a national level, our study has several limitations. First, MRONJ was mainly classified using the ICD code K10.2 without considering the clinicopathological aspects. A code for more specific pathological features for MRONJ (M87.1) was introduced in 2010, but many physicians and dentists were not familiar with this new code to describe the osteonecrosis of jaw after BMA treatment at the beginning of the code change. Bergdahl et al. reported that osteonecrosis due to drugs (M87.1) had the highest positive predictive value (83%; 95% confidence interval, 36–100%) in a study using data from the Swedish National Patient Registry. Inflammatory conditions of the jaw (K10.2) had a low positive predictive value (16%) [23]. Therefore, we also analyzed codes M87.1 and K10.2, but there was no significant difference between M87.1 and K10.2 in our database. Second, we didn’t collect the individual dental treatment history before BMA treatment and distinguished whether systemic complications such as sepsis were related to the use of BMA. We found this kind of information was difficult to extract relevant data exactly using the national claims database system. Third, medical doctors conventionally used to prescribe pain killers or empirical antibiotics rather than refer them to the dentist. These factors may contribute to the low reported dental screening and AEs frequency during BMA treatment. Fourth, this study found no significant difference in the overall incidence of AE between patients who underwent dental check-ups before BMA treatment and those who did not. Moreover, we did not specifically examine the incidence of MRONJ between these two groups. Consequently, our findings do not provide direct support for the hypothesis that pre-treatment dental examinations prevent spontaneous MRONJ or reduce the rate of tooth extraction after treatment initiation. Further research is necessary to explore these hypotheses in greater depth.

Despite these limitations, this is the first nationwide study to investigate the prevalence of dental check-ups and a wide range of dental AEs including MRONJ before and after BMA treatment. Based on this information, cooperation between oncologists and dentists to prevent dental AEs can be started and expanded to build up preventive practice for the dental care plan. In patients who initiate a BMA, preventive care includes comprehensive dental assessments, discussion of modifiable risk factors, and avoidance of elective surgery during BMA treatment. By establishing a personalized dental care plan, we will be able to satisfy the unmet needs of patients with bone metastasis breast and prostate cancer who need long-term chemotherapy and BMA treatment.

### Electronic supplementary material

Below is the link to the electronic supplementary material.

Supplementary Material 1

## Data Availability

All data presented in this study are owned by the authors. No third-party material is included in the manuscript, and thus, no permissions are required.

## Abbreviations

BMA: Bone-Modifying Agents
MRONJ: Medication-Related Osteonecrosis Of The Jaw
