# Supplementary material for: The trend of dental check-up and prevalence of dental complications following the use of bone modifying agents in patients with metastatic breast and prostate cancer: analysis of data from the Korean National Health Insurance Service
Source: BMC Health Serv Res. 2024 Apr 2;24:412. doi: 10.1186/s12913-024-10859-7 (PMC10988945; doi:10.1186/s12913-024-10859-7)
Supplement: Supplementary file 1 — Supplementary Material 1 [file 12913_2024_10859_MOESM1_ESM.docx]

**Additional Table 1.** The occurrence of patients with bone metastasis from breast and prostate cancer treated with BMAs

| Year | *N* (%) |
| --- | --- |
| 2007*  2008  2009  2010  2011  2012  2013  2014  2015  2016  2017  2018  2019 | 480 (3.1)  1,015 (6.5)  1,164 (7.5)  1,142 (7.4)  1,179 (7.6)  1,297 (8.4)  1,306 (8.4)  1,274 (8.2)  1,291 (8.3)  591 (3.8)  1,710 (11.0)  1,773 (11.4)  1,292 (8.3) |
| Total | 15,357 (100.0) |

* Patients who were prescribed BMA before July 1, 2007 due to wash out period (6 months)

*BMA*, Bone-modifying agent

**Additional Table 2.** Time to occurrence of MRONJ by BMAs

|  | MRONJ occurrence, *N* (%) | Mean (SE),  days | p-value* |
| --- | --- | --- | --- |
| BMA, *N* (%)  Zoledronic acid  Pamidronate | 51 (91.1%)  5 (8.9%) | 2396.36 (1.40)  1306.91 (0.62) | <0.0212 |

* p-value by Log-rank test

MRONJ, medication-related osteonecrosis of the jaw; *BMA*, Bone-modifying agent; SE, standard error.


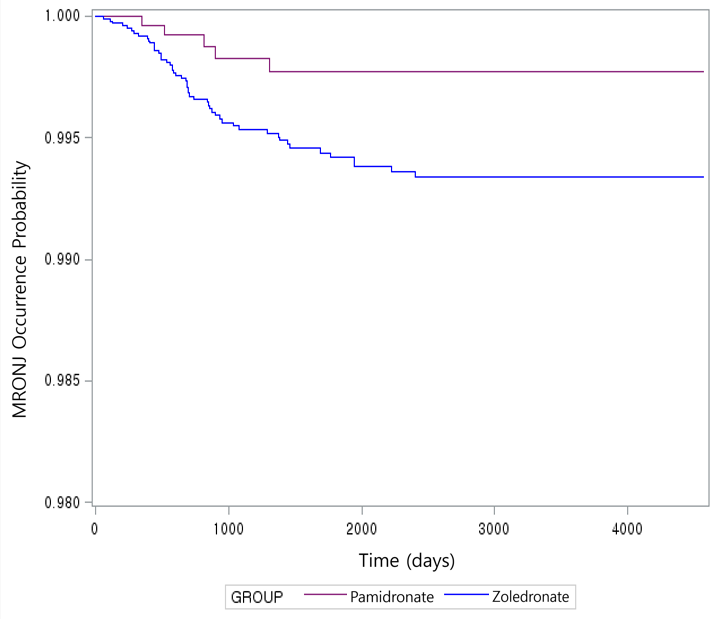


**Additional Fig. 1** Time to occurrence of MRONJ by BMAs

MRONJ, medication-related osteonecrosis of the jaw; *BMA*, Bone-modifying agent
